# Ophthalmologic Manifestations of Primary Sjögren’s Syndrome

**DOI:** 10.3390/genes12030365

**Published:** 2021-03-04

**Authors:** Anna Maria Roszkowska, Giovanni William Oliverio, Emanuela Aragona, Leandro Inferrera, Alice Antonella Severo, Federica Alessandrello, Rosaria Spinella, Elisa Imelde Postorino, Pasquale Aragona

**Affiliations:** 1 Ophthalmology Clinic, Department of Biomedical Sciences, University Hospital of Messina, 98124 Messina, Italy; giowino89@gmail.com (G.W.O.); inferreraleandro@gmail.com (L.I.); aliceantonellasevero@gmail.com (A.A.S.); fedeale90@gmail.com (F.A.); s.sara.spinella@gmail.com (R.S.); elisapostorino@virgilio.it (E.I.P.); paragona@unime.it (P.A.); 2 IRCCS San Raffaele Scientific Institute, Ophthalmology Clinic, Vita Salute San Raffaele University, 20132 Milan, Italy; aragona.emanuela@hsr.it

**Keywords:** Primary ocular Sjögren’s syndrome, pathogenesis, innate immunity, adaptive immunity

## Abstract

Sjögren’s syndrome (SS) is a chronic, progressive, inflammatory, autoimmune disease, characterized by the lymphocyte infiltration of exocrine glands, especially the lacrimal and salivary, with their consequent destruction. The onset of primary SS (pSS) may remain misunderstood for several years. It usually presents with different types of severity, e.g., dry eye and dry mouth symptoms, due to early involvement of the lacrimal and salivary glands, which may be associated with parotid enlargement and dry eye; keratoconjunctivitis sicca (KCS) is its most common ocular manifestation. It is still doubtful if the extent ocular surface manifestations are secondary to lacrimal or meibomian gland involvement or to the targeting of corneal and conjunctival autoantigens. SS is the most representative cause of aqueous deficient dry eye, and the primary role of the inflammatory process was evidenced. Recent scientific progress in understanding the numerous factors involved in the pathogenesis of pSS was registered, but the exact mechanisms involved still need to be clarified. The unquestionable role of both the innate and adaptive immune system, participating actively in the induction and evolution of the disease, was recognized. The ocular surface inflammation is a central mechanism in pSS leading to the decrease of lacrimal secretion and keratoconjunctival alterations. However, there are controversies about whether the ocular surface involvement is a direct autoimmune target or secondary to the inflammatory process in the lacrimal gland. In this review, we aimed to present actual knowledge relative to the pathogenesis of the pSS, considering the role of innate immunity, adaptive immunity, and genetics.

## 1. Introduction

Sjögren’s syndrome (SS) was described first in 1933 by a Swedish ophthalmologist, Henrik Sjögren. SS represents a chronic, progressive, inflammatory, autoimmune disease, characterized by the lymphocyte infiltration of exocrine glands, especially the lacrimal and salivary, with their consequent destruction [1,2,3,4].

SS may present as primary (pSS) or secondary (sSS) to connective tissue disorders, such as lupus erythematosus, rheumatoid arthritis, systemic sclerosis and, less frequently, multiple sclerosis, thyroiditis, and autoimmune hepatitis [5,6].

The prevalence of pSS varies between 0.05% and 4.8% in the literature, and several studies have reported different prevalence rates of the disease, in relation to the geographical area considered, and the criteria used to make the diagnosis [7,8,9,10,11]. As the majority of autoimmune diseases, it is more frequent in females, showing two peaks of incidence, the first between 20 and 40 years, and the second after menopause.

The onset of pSS may remain misunderstood for several years. It usually presents with dry eye and dry mouth symptoms due to early involvement of the lacrimal and salivary glands, which may be associated with parotid enlargement [1,12,13]. In more than 30% of cases, systemic manifestations may occur, with involvement of the kidneys, lungs, skin, joints, and muscles [12].

### 1.1. Signs and Symptoms

Ocular signs and symptoms are represented by surface disorders with different severity grades. Patients claim foreign body sensations, reduced or increased tearing, itching, and blurred vision. The slit lamp examination shows redness, conjunctival keratinization with chalasis, and punctate or filamentous keratitis [14,15]. In some cases, the seborrheic blepharitis can be evidenced [16].

The symptoms may be exacerbated by conditions that reduce blinking rate, such as prolonged reading or use of electronic devices. If not adequately treated, the ocular changes may lead to severe complications, such as corneal melting, perforation, and bacterial infection with a risk for the visual function [17].

Salivary gland involvement appears with xerostomia. Patients refer to difficulty in chewing and swallowing, and alterations in taste and smell. In the physical examinations, the mucous membranes appear dry and there may be skin cracks in the corners of the mouth [18]. The biopsy of minor salivary glands confirms diagnosis with sensitivity ranging from 63.9 to 85.7% and specificity of 61.2 to 100%. A lymphocyte infiltrate with aggregates of 50 elements on 4 mm^2^ is called lymphocytic sialadenitis, and is compatible with SS. However, the histological negativity does not exclude the diagnosis [19].

Involvement of the other exocrine glands may present with chronic bronchitis, nasal mucosal dryness, recurrent pneumonia, acute pancreatitis, liver disease, vaginal mucosal dryness, or dyspareunia [15,20]. Extra-glandular manifestations, such as joint deformities, interstitial pneumonia, renal and neurological involvement, and asthenia could be associated with the typical symptoms [21]. Patients with SS have a 6-fold increased risk of developing non-Hodgkin’s lymphoma. In fact, in these patients, hyperactivity of B cells with an increase in the B/T cell ratio were highlighted. The consequent formation of germinal center-like aggregates was associated with the risk of developing lymphomas [22].

### 1.2. Diagnosis

Several criteria have been considered to diagnose SS over the past 50 years; in the last 10 years, the most used were those of the American–European Consensus Group (AECG) [3]. In 2012, the American College of Rheumatology (ACR) validated criteria provided by data from the SICCA register (Sjögren’s International Collaborative Clinical Alliance), and while the previous are based on the ocular and oral symptoms, to classify patients, the latter rely on the objective testing. In 2012, a joint ACR/EULAR consensus redefined the classification criteria for primary Sjögren’s syndrome [23].

Moreover, SS classification criteria are based on the weighted sum of five items: anti-SSA/Ro antibody positivity, focal lymphocytic sialadenitis with a focus score ≥1 foci/4 mm^2^, ocular staining score (≥5) or van Bijsterveld score (≥4), Schirmer test ≤5 mm/5 min, and unstimulated salivary flow rate ≤0.1 mL/min [24].

These criteria apply to patients who present at least one symptom of dry eye or mouth and do not have exclusion criteria, such as hepatitis C infection, HIV, sarcoidosis, amyloidosis, or head and neck radiotherapy. Patients with sicca symptoms (for at least 3 months) require further diagnostic investigations [24].

Several studies attempted to find biomarkers that could be used in SS diagnosis. Some of them, such as cytokines and autoantibodies, have been detected in the serum, and the others in the DNA or in the cellular structures [22]. The serum biomarkers are the most used and comprise anti-Ro/SSA and anti La/SSA antibodies, antinuclear antibody [14], antibodies directed towards muscarinic receptors (M3R) [22], and anti-rheumatoid factor antibodies [22].

New emerging molecules, such as profilin and carbonic anhydrase I (CA-I), which could represent a valid tool to avoid invasive diagnostic procedures, such as biopsy, were evidenced in saliva and tears [25]. Additionally, an increase in IL-4 and IL-5 together with clusterin, an indicator of inflammation, proved to be a good predictor of the disease in 93.8% of cases [25]. Increase in the cathepsin S level in tears was considered a useful biomarker of SS [25,26].

Other studies focused on different molecules, such as FMS-like tyrosine kinase 3 ligand (Flt-3L) [27,28], beta2-microglobulin, free light chains of immunoglobulins [26,29], and siglec-1 [26,30]. Interesting results were obtained with genomic biomarkers, such as IFN type I-inducible genes and microRNAs. In fact, IFN type I-inducible genes increased in the peripheral blood and salivary glands of subjects with pSS and their presence was related to the higher levels of activity of the disease [25,31,32,33].

MicroRNAs were correlated to inflammatory changes in the salivary glands of patients with SS and their identification in saliva through PCR allowed their use as biomarkers.

An increase of miR-146a was detected in subjects with pSS and the presence of miR-768-3p and miR-574 was associated with a reduction in glandular inflammation [25,28,34,35].

For all of these reasons, IFN type I-inducible genes and microRNAs could represent an important diagnostic tool and therapeutic target in the next future and could provide information about the activity of the disease.

Despite these findings, to date, there is no pathognomonic biomarkers of Sjögren’s syndrome and biopsy of the minor salivary gland remains the gold standard for diagnosis. Salivary gland ultrasonography (SGUS), a non-invasive method used to study the structure of the parotid and submandibular glands provides information related to the subversion of the glandular structure and the presence of fibrosis or calcifications [1]. Based on the ultrasound inhomogeneity of the glandular tissue, many scores were created to classifying the degree of the disease (0–3 scoring system, 0–4 scoring system, 0–16 scoring system, 0–48 scoring system). Each score has its own advantage and, to date, the most reliable score is being debated. The 0–4 score system showed the 75% sensitivity and 93% specificity, and it is used as a universal SGU diagnostic standard with a higher specificity and less heterogeneity than the other scoring systems [36]. SGU was recommended as a diagnostic tool as it was demonstrated that the results of ultrasonography are comparable to those of sialography [36,37,38].

Presently, histological investigation plays a key role in the diagnosis of SS and the biopsy of the labial salivary glands (LSG) remains as the main method, due to its high specificity with minimal invasiveness. Histologically, it is possible to evidence the presence of agglomerates of T and B lymphocytes surrounding the ducts and small vessels [22]. A set of 50 monocytic cells/4 mm^2^ was defined as a focus score (FS); a FS ≥1 correlates with SS [14].

## 2. Pathogenetic Mechanisms

Recent scientific progress in understanding the numerous factors involved in the pathogenesis of pSS was registered, but the exact mechanisms involved still need to be clarified [10,11].

The role of both the innate and adaptive immune system in actively participating in the induction and evolution of the disease is unquestionable [39].

### 2.1. Innate Immunity

Activation of the innate immune reaction and the consequent secretion of interferons (INFs) due to an environmental trigger is involved in the first phase of pSS pathogenesis [39].

There is a paucity of data about the early pathogenic process; however, a viral infection involving the epithelial cells of exocrine glands—such as Epstein–Barr virus (EBV), cytomegalovirus (CMV), HIV, hepatitis C virus (HCV), coxsackievirus, human herpesvirus type 8 (HHV-8), and human trophic lymphocyte virus type 1 (HTLV-1)—is the most accredited hypothetical trigger factor of the initial autoimmune response, in addition to a specific genetic and environmental background [39,40,41,42].

In this stage, toll-like receptors (TLRs), involved in the detection of pathogen-associated molecular patterns, play a crucial role in the innate immune system. Indeed, their activation, in response to a viral infection or immune complex formation, represents the opening of an innate immunity reaction, and this recognition leads to the upregulation of the type 1 IFN pathway [39].

The importance of these receptors was confirmed using the NZB/W F1 mouse model, one of the most studied rodent models for pSS, as the treatment with the TLR3 agonist poly(I:C) leads to inflammation of the salivary glands, typical for pSS, through the activation of the innate immune system [43]. Moreover, the innate reaction produces dendritic cell (DC) and epithelial cell activation; in particular, activated acinar and ductal epithelial cells of exocrine glands act as non-professional antigen presenting cells (APCs), expressing autoantigens and a variety of immunomodulatory molecules involved in the recruitment and stimulation of several immune cells [39,41,42,43,44].

The apoptosis of these cells is an essential moment in the expression of autoantigens, such as Ro/SSA and La/SSB. Actually, the control of apoptosis of salivary gland epithelial cells represents another crucial point in the pathogenesis of pSS, including several factors, such as the Fas/Fas ligand system, the tumor necrosis factor related apoptosis-inducing ligand (TRAIL) and TLR3 [39].

Activated epithelial cells secrete fundamental cytokines, such as IL-21 and B cell activating factor (BAFF) induced by type I and type II interferons, which drive the activation and differentiation of T and B cells [39,41,42,43,44,45]. A high level of BAFF was also found in the salivary gland of patients with pSS, and it was correlated with increased disease activity [45]. For these reasons, the innate immune activation leads to an autoimmune response by the adaptive immune system.

### 2.2. Adaptive Immune System

Recent studies highlighted the multiple roles of B cells in pSS pathophysiology, playing a central role in the loss of immune tolerance and in the development of the disease [39,42]. These cells are mainly responsible for immunoglobulins and antibody secretion, as well as for the antigen presentation process [42]. Moreover, B cells realize additional functions, acting as APCs, and producing cytokines that can sustain the immune response [39,42]. In patients with pSS, peripheral blood and salivary gland B cell subset distribution is altered, leading to the constitution of ectopic germinal centers (GC) where autoreactive clones may escape tolerance checkpoints [39]. The presence of germinal centers is described in the exocrine glands in patients with pSS. These are essential for the differentiation and proliferation of specific B cells, through the expression of specific homing molecules, such as C-X-C motif chemokine 5 (CXC5) and its ligand CXCL13, principally expressed by follicular helper T (T_FH_) cells, fundamental for migration into the B cell zone of secondary lymphoid organs [42].

On the other hand, the correlation between pSS and several major histocompatibility complex class 2 (MHC2) alleles were demonstrated, suggesting that autoantigen presentation is essential in the pathogenesis of the disease [39]. Increased levels of pro-inflammatory T_H_1 cytokines, such as IL-1b, IL-6, TNF-α, and IFN-γ, were found in patients with pSS in tears, conjunctiva, lacrimal, and salivary gland and blood, demonstrating the higher prevalence of a T_H_1 cells response [46]. Furthermore, in the non-obese diabetic (NOD) mouse, a conventional model of pSS, the absence of IFN-γ, or its receptor expression abolished development of the disease [47,48]. Finally, the level of IFN-γ is also correlated with the severity of the disease [49]. IL-12 is a fundamental inducer of T_H_1 response, contributing to the secretion of IFN-γ. Furthermore, IL-12 is involved in the cross-talking mechanism between DCs and natural killer (NK) cells [50]. For these reasons, IL-12 seems to be a central cytokine in pSS pathogenesis, as further suggested by the rodent model that overexpresses IL-12, manifests a pSS-like syndrome [51,52]. Recently, increased amounts of T_H_17 cells were also discovered at sites of inflammation in salivary gland biopsies, and in serum of pSS patients, highlighting the potential role of T_H_17 response in the pathogenesis of pSS [39,53].

T_H_17 cells are able to produce pro-inflammatory cytokines, such as IL-6, IL-17, IL-22, and IL-23, where increment is determined within pSS salivary glands. Moreover, the expression of IL-17 by these cells is involved in the maintenance of the inflammatory process, and is related to the severity of pSS [39,53].

NK cells exhibit the potentiality to downregulate T cell responses. Recent studies demonstrated the presence of peculiar immune cells in the salivary glands of mouse, tissue-resident memory T cells (TRM cells) that are antigen-experienced T cells, commonly situated in non-lymphoid organs, and NK1.1+ cells, a unique kind of cell population of innate lymphoid cells (ILC) with characteristics of both conventional NK cells and ILC1 [54,55].

Additionally, recent papers demonstrated the fundamental role of resident macrophages in salivary glands to repair and restore salivary function after damage through the secretion of pro-repair factors [56,57].

### 2.3. Genetics

SS can be considered the result of the interaction between environmental and genetic factors. In the past few years, labial salivary gland and peripheral blood gene expression microarray studies focused on genetic components of SS susceptibility. Several single nucleotide polymorphisms contribute to increased susceptibility to SS. These include regulation of the innate immune system through type-1 interferon (IFN) axis; B cell (and T cell) trafficking mediated by C-X-C motif chemokine receptor 5 (CXCR5)-driven immune cell recruitment to ectopic B cell follicles; B cell lymphocyte kinase mediated B cell receptor activation; IL-12-IFN-γ cell receptor activation; and IL-12-IFN- γ axis Th1-related pathway [58].

Additionally, a strong association with human leukocyte antigen (HLA) molecules, including HLA-DR, HLA-DQB1, and HLA-DQA1, was determined. Lessard et al. established a strong association with the HLA region at 6p21, and found IRF5-TNPO3, STAT4, IL12A, FAM167A-BLK, DDX6-CXCR5, and TNIP1 as at-risk loci [59].

Genetic at-risk loci contribute significantly to the development of pSS. Scientific evidence demonstrated that epigenetics alterations might play an important role in the pathogenesis of the disease. DNA methylation regulates the transcriptional accessibility of a gene; in particular, the hypomethylation of the promoter allows the active transcription, whereas methylation on a gene promoter leads to silencing the gene expression. In pSS, the hypomethylation of IFN-regulated genes (MX1, IFI44L, PARP9) in the B cells (CD19+) in minor salivary glands was found with an increase of the gene expression [60]. Other dysregulate immune pathways correlated to interferon signaling and comprised the genes IRF5, STAT4, IL12A, and OAS1. Regarding to the B cell function, EBF1, BLK, FAM167A, TNFAIP3, and TNIP1were established as genes with risk alleles [61].

MicroRNAs regulate the post-transcriptional gene expression where the complete match between miRNA and 3′ untranslated region of the target mRNA sequence induces mRNA degradation whereas the incomplete match prevents mRNA from being translated. Salivary gland tissue and peripheral blood mononuclear cells analysis revealed the differential miRNA expression patterns, especially for T and B cells. Twenty-six miRNA with aberrant expression patterns in peripheral blood cells were found. The differential expression patterns for miRNA were associated with IRF5, STAT1, and IRAK1 in blood derived T cells and B cells. In particular, IRAK1 appears downregulated, due to transcriptional repression by miR-146a, which resulted increased. In SS patients, miRNA associated with the B lymphocytes survival pathways, including PI3K-PKB signaling pathway and BAFF, are differentially expressed. In particular, miR-30b-5p was identified as a negative regulator of BAFF. In CD4^+^ T cells, downregulation of miR-let-7d-3p, miR-30c-5p, miR-378a-3p, and upregulation of miR-155-5p, miR-222-3p, miR-146a-5p, and miR-28-5p were identified. In CD19^+^ B cells, miR-378a-3p, miR26a-5p, miR30b-5p, miR-19b-3p appeared to be reduced, and miR-222-3p was upregulated [60,62]. The study of miRNA is an emerging field, and its role in the pathogenesis of SS is still to be defined.

### 2.4. Hormonal Influences

Hormonal influences have been widely described in SS, as the disease is more prevalent in females than males, particularly in post-menopausal age [63]. These differences are certainly linked to the effects of sex steroids, such as androgens and estrogens. Indeed, numerous studies demonstrated the influences of these hormones on the ocular surface structures and function, acting on the Meibomian gland, lacrimal gland, conjunctival, and corneal epithelium [63,64]. Sex steroids exert a significant influence on the cellular architecture, gene expression, protein synthesis, and fluid and protein secretion [63].

Testosterone is the principal circulating androgen in men, while estrogen and progesterone are present in minor quantities. In reproductive-age females, 17-β-estradiol (E2) and progesterone are the most abundant sex steroids; however, testosterone, dehydroepiandrosterone (DHEA), and androstenedione are also present. After menopause, even if the amount of estrogen produced by the ovaries is reduced, the production of estrogens is maintained by intracrine processes that allow the peripheral transformation of DHEA into estrogen thanks to steroidogenic enzymes present in the peripheral tissues. Throughout the years, the DHEA pool changes, even if, after menopause, the ovarian contribution to the production of testosterone increases production by the adrenal glands decrease, resulting overall in a net decline [65,66,67]. These sex-steroid imbalances contribute to female predominance and age of onset of SS [63]. In fact, lower levels of the sex-steroid precursors DHEA and DHEA-S were reported in the blood and saliva of SS patients as compared to healthy subjects [68,69]. Moreover, androgen deficiency has been associated to the inflammatory reaction of the lacrimal gland in SS, as well as ocular surface damage and tear film instability [70]. The role of testosterone was investigated in lacrimal glands of both MRL/lpr and NOD mice, showing that the majority of immune-response genes regulated by testosterone were of the inflammatory type [71].

However, previous studies of the effect of DHEA oral supplementation in post-menopausal SS patients showed controversial results [70,72]. Conversely, the exact role of estrogen in the physiology and pathophysiology of ocular surface is still uncertain and debated [63,70,72]. Additionally, hormonal influence on immune system activity is reported [73]. In fact, adult females demonstrated stronger innate and adaptive immune response than man, and this could be at the basis of their increased predisposition to autoimmune disorders [73].

Some differences may be germline encoded, such as the higher expression of TLR7 in females [74]. Furthermore, sex steroids influence the production of cytokines and chemokines by innate immune cells, as well as APC activity [73,75]. Moreover, sex differences were reported in innate-like lymphocytes that regulate numerous tissue immune response [76].

Concerning the adaptive immunity, females have higher CD4^+^ T cell counts and higher CD4/CD8 ratio than males [77]. E2 levels could exert different actions on the immune system, enhancing both humoral and cell-mediated immune response. Usually, low levels of E2 promote T_H_1 and cell-mediated response, while high levels of E2 lead to T_H_2 and humoral response [78]. The immunosuppressive action of androgen is well descried. Indeed, testosterone and DHT induce IL-10 and TGF-β, leading to an anti-inflammatory response [73,79,80]. All of these differences described could explain the differences in incidences of autoimmune diseases, such as SS.

## 3. Ocular Surface Unit Involvement

Dry eye or keratoconjunctivitis sicca (KCS) is the most common ocular manifestation of pSS [81].

It is still doubtful if the extent ocular surface manifestations are secondary to lacrimal or meibomian gland involvement or to the targeting of corneal and conjunctival autoantigens [81]. However, Sjögren’s syndrome is the most representative cause of aqueous deficient dry eye, and several in vivo and in vitro studies confirmed the primary role of the inflammatory process involving the lacrimal gland and the ocular surface, suggesting several crucial targets for treatment [11].

### 3.1. Lacrimal Gland

The autoreactive damage of the lacrimal gland represents the hallmark of the ocular involvement of the disease; however, our knowledge of the destructive inflammatory process is mainly deduced from histopathologic studies of minor salivary glands and from the mouse models of pSS [82].

The rodent model NZB/W F1 developed autoimmunity characterized by lymphocytes B hyperreactivity and autoantibodies production, and showed lymphocytic infiltration of the lacrimal glands, initially with foci pattern, which progress with alterations of the acinar architecture and consequent sicca syndrome development [83].

The pathologic lesions described in the salivary gland are characterized by an infiltration of clusters of round cells, where composition depends on the severity of the lesion. CD4^+^ T cells prevail in mild lesions, while CD8^+^ T cells and B cells in severe forms [11,82]. Furthermore, other infiltrative cells, such as macrophages and dendritic cells, are observed in the exocrine glands, being more present the milder lesions [84]. The grade of cellular infiltration is correlated to the lacrimal gland function, as a reduced tear reflex secretion related with the presence of SS autoantibodies [85,86,87,88,89]. The histopathological hallmark of SS is represented by periductal and perivascular inflammatory infiltration of lymphocytes in the salivary and lacrimal glands, which is responsible for the total damage of acini, with a loss of the gland secretory function and, in some cases, development of B cell lymphomas. Recent studies on a mouse model demonstrated that, in the course of SS, early inflammation occurs concomitantly in the submandibular and lacrimal glands, with a late (and less severe) involvement of the parotid glands, rare of the sublingual glands [90].

Together with the destruction of glandular tissue, due to the infiltration and proliferation of lymphocytes, the establishment of ectopic GC-like was observed in a significant percentage of pSS patients [91]. Furthermore, the architectural alterations in the salivary gland are supplemented with the growth of adipose tissue and fibrosis [92]. Numerous characteristics of the lacrimal gland alterations in pSS were extensively evaluated in the TSP-1 -/- model, and the progressive chronic inflammation leads to structural, functional, and neurological damage that impacts the secretory function of the lacrimal gland [93]. Lymphocytes, DCs, activated epithelial cells, and neural cells are able to activate and maintain the inflammatory reaction, by releasing cytokines and pro-inflammatory mediators, resulting in apoptosis and loss of secretory acinar cells and ductal damage, and, consequently, aqueous tear film reduction [39,93]. Furthermore, the inflammatory damages lead to a secretomotor innervation injury, as well as to the inhibition of neurotransmitter release or action by cytokines or antibodies, which could reduce the gland secretion [39].

The non-obese diabetic mouse models (variant strain NOD.B10.H2b) demonstrated numerous similar features in the lacrimal glands in pSS subjects, comprising lymphocytic infiltrations and the defeat of secretory functions [94].

The lacrimal gland with its efferent nerves and the afferent nerves of the conjunctiva and cornea constitutes a functional unit that secrete the aqueous component of the tear film. As the tear reflex is related to the corneal nerve terminals, both the density of sensory nerve fibers, and their functions, were found reduced in pSS patients [95]. Indeed, corneal nerve alterations in the early phase of pSS have been reported using in vivo confocal microscopy and could be a preliminary sign of SS-related dry eye disease [96]. Furthermore, alterations in the lacrimal gland morphology of secretory vesicles, and in the distribution of key effectors of exocytosis, indicating the primary involvement in the lacrimal and submandibular glands, are in agreement with the human studies [90].

Moreover, the Aec and IL-12 transgenic mice both demonstrated T cell infiltration in the exocrine glands; TSP-1KO mice, which presented anti-SSA and anti-SSB autoantibodies in serum, showed modifications in the ocular surface microbiota; Cd25 knockout mice presented altered immune tolerance mechanisms, leading to lacrimal gland destruction and goblet cell loss [52,97,98,99].

### 3.2. Keratoconjunctival Involvement

The ocular surface inflammation is a central mechanism in pSS leading to the decrease of lacrimal secretion and keratoconjunctival alterations [81]. However, there are controversies about whether the ocular surface involvement is a direct autoimmune target or secondary to the inflammatory process in the lacrimal gland [11]. The main knowledge about the conjunctival involvement in pSS derives from studies on impression cytology, which provide data about the epithelium status [11]. These studies documented the lymphocytic infiltration of the conjunctiva in patients with pSS, which were prevalently CD4^+^ T, and in a lower quantity of CD8^+^ T cells and B cells, respectively [38,100].

Furthermore, the expression of HLA-DR and HLA-DQ has been demonstrated, not only by lymphocytes, but also by conjunctival epithelial cells, suggesting a possible role for them as non-professional APCs [11]. Likewise, the presence of ICAM-1, a cell surface adhesion molecule fundamental for the lymphocyte homing during the inflammatory process, was demonstrated on epithelial cells, as well as the expression of LFA-1, its T cell ligand, was identified [101,102,103]. The interaction between epithelial cells and T cells through the ICAM-1 molecule is implicated in the apoptotic process of the epithelial cells [101,102,103].

In fact, all of these pieces of evidence documented the central role of the inflammation in the ocular surface involvement in patients with pSS, as the increased level of pro-inflammatory cytokines has been discovered on the ocular surface and in tears; in particular, TNF-α, IL-1α, IL-1β, 1β, IL-6, and IFN-γ were commonly found in these subjects [104,105]. Moreover, a study showed that patients with pSS had higher IL-17, IFN-γ, and lower MUC5AC mRNA transcripts when compared to normal subjects [106]. Indeed, the morphological changes observed in the corneal–conjunctival epithelial cells could be related to these pro-inflammatory factors, and combined with the inflammation in the lacrimal gland, and reduced concentration of essential lacrimal gland-derived factors, such as epidermal growth factor (EGF), could create an environment in which terminal differentiation of the ocular surface epithelium is impaired [107].

Furthermore, the higher level of MMP-9 and TG-2 was found in patients with pSS, compared to subjects with evaporative dry eye disease, as a result of the direct autoimmune insult to the ocular surface epithelia [108]. The loss of goblet cells is a crucial feature of the ocular surface involvement in pSS. Indeed, increased expression of the T_H_1 cytokine and IFN-γ, which induce an unfolded protein response and apoptosis in goblet cells, has been found in KCS [11]. Goblet cells are fundamental in the homeostatic process of the ocular surface, through the release of mucins that lubricate and protect the corneal–conjunctival epithelium, as well as producing immunomodulatory factors, such as TGF-β2, mucin 2 (MUC2), retinoic acid, fundamental for maintaining the immune tolerance, typical of a healthy ocular surface [109]. In fact, goblet cells through the production of transforming growth factor-β (TGF-β), participate in the immune tolerance process by selecting tolerogenic DC, able to stimulate the homing of regulatory T cells (T-regs) on the ocular surface [11].

In pSS, the chronic inflammation is responsible for the squamous metaplasia that involves the corneal–conjunctival epithelial cells, due to the modification of the glycocalyx, dropout of goblet cells, and keratinization [11]. High levels of IFN-γ and IL-1β playing a fundamental role in the squamous metaplasia process of the corneal–conjunctiva epithelium [11]. IL-1β shows a pro-inflammatory activity, leading to a release of IL-6, IL-8, TNF-α on the ocular surface [110]. Furthermore, IL-1β has been associated with the keratinization process that involve the ocular surface, as demonstrated by the marker expression SPRR1B of squamous metaplasia [111].

IFN-γ released by T_H_1 cells and NK cells, also plays a fundamental role in this process; in fact, it stimulates the apoptosis and goblet cell dropout of the corneal–conjunctival epithelium and keratinization process in the severe KCS [108]. IL-1β and IFN-γ are also able to stimulate the expression of small proline-rich proteins (SPRRs), involved in the squamous metaplasia process, as demonstrated by in vitro studies, through the triggering of the p38 MAPK pathway [112].

Previous studies demonstrated that the number of conjunctival APCs correlated with categorical clinical severity and inversely correlated with the number of goblet cells [113]. Nowadays, confocal microscopy, a relatively new instrument allows to identify inflammatory cells, goblet cells, and corneal sub-basal nerve plexus fibers in vivo. Numerous confocal microscopy studies revealed changes in corneal epithelium in pSS patients, demonstrating a reduced cell density of the inner layer of wing cells, as well as the density of basal epithelial cells, increased conjunctival epithelial microcyst density, and increased conjunctival inflammatory cell density [114,115]. Benitez-del-Castillo JM and coworkers, reported an increase of corneal anterior keratocytes density in pSS patients, compared with the healthy control subjects, as a result of chronic inflammation induced by the pro-inflammatory cytokines released, leading to activation of keratocytes. The activated keratocytes, characterized by a hyperreflective appearance at the confocal microscopy, produce and secrete nerve growth factor (NGF), acting in the process of reorganization of the sub-basal nerve plexus in patients with SS [116,117]. In fact, the corneal sub-basal epithelial nerve number and density were found reduced in patients with SS, when compared with the healthy control subjects [116,117,118]. Furthermore, the exalted inflammatory mechanism and the release of IL-1 and TNF-α are responsible for apoptosis and increased proteolytic activity, leading to corneal thinning of the stroma [11].

### 3.3. Meibomian Glands

A high prevalence of Meibomian gland dysfunction has been evidenced in patients with pSS [119]. Previous studies have demonstrated that patients with pSS showed reduced Meibomian gland secretes quality, gland expressivity, and gland dropout [120,121]. Moreover, a reduced thickness, stability, and surface activity of the lipid layer of tears, leading to an increased evaporability were reported; indeed, the presence of a clinical combination of tear deficiency and increased evaporation DED is common in patients with pSS [81,121,122]. Meibomian gland dropout in patients with pSS was found as more severe than in non-SS DED patients [123,124]. Furthermore, Sullivan et al. demonstrated that women with pSS showed a higher prevalence of occluded and metaplastic Meibomian gland orifices, and reduced quality of their secretions, characteristic of obstructive Meibomian gland dysfunction MGD [119]. The authors hypothesized that one of the main mechanisms contributing to MGD in women with pSS is the androgen deficiency [119].

Secondly, the conjunctival inflammation with the lymphocyte accumulation leads to tarsal and peri-glandular inflammation; thus, creating a toxic environment. The release of proinflammatory cytokines affect the terminal duct of the Meibomian gland with the aforementioned sequelae [119].

Moreover, confocal microscopy studies demonstrated higher reflectivity and inflammation near the meibomian gland in patients with SS more accentuated than in patients without SS [96].

## 4. Therapy

Therapy of the primary SS comprise both a topical and systemic approach in relation to the severity of the disease. Additionally, the punctal plugs might be used to reduce tear outflow and maintain the film volume. However, their insertion causes an accumulation of a pro-inflammatory protein, for this reason, the usage is controversial [125].

### 4.1. Topical Therapy

Topical therapy with teardrops and ocular gels is considered the first line treatment for dry eye disease (DED) associated to the pSS, aiming to increase the volume of the tear film and to reduce the friction between the lid and ocular surface [126].

The most common tear substitutes contain sodium hyaluronate (HS) with different concentrations, and several studies demonstrated its clinical efficacy with improvement of symptoms and ocular tests in patients with DED [127,128,129,130,131,132]. The use of non-steroidal anti-inflammatory (NSAIDs) drops was considered in the therapy for the ocular surface alterations in SS patients. It was demonstrated that the NSAIDs reduce the ocular discomfort, but, at the same time, might induce the ocular surface adverse effects [81,133,134]. Topical corticosteroids proved effective in improvement of the ocular surface signs and symptoms; however, the onset of adverse effects should always be considered and monitored [135,136]. The use of cyclosporine A (CsA) [137,138,139,140,141,142,143] and autologous serum [144,145] platelet rich plasma (PRP) [146,147] in pSS-DED was studied, but contrasting results were reported. Other treatments, such as mesenchymal stem cells or multipotent stromal cells (MSCs) are in the experimental phase, but have shown promising results, demonstrating immunomodulation and wound healing ability [148,149,150,151,152,153,154]. Implantation of MSCs in the dd mouse and canine model highlighted encouraging results in tear film secretion, tear film stability, and epithelial cells/goblet cells regeneration [155,156,157].

### 4.2. Systemic Therapy

As to the systemic therapy, the pilocarpine, a muscarinic cholinergic parasympathomimetic agonist, which stimulates exocrine glands secretion by binding muscarinic (M3) receptors, is actually the principal choice. In fact, the formulation for oral use showed significant clinical efficacy in improving dry eye symptoms [158,159,160,161,162]. The use of other principles, such as cevimeline hydrochloride, a muscarinic acetylcholine agonist, demonstrated clinical efficacy, but different adverse effects limiting its use were registered [163,164,165].

Interesting results were obtained with rituximab, a monoclonal antibody, directed against the CD20 protein and expressed on mature B cells and hydroxychloroquine, an immunomodulator that interfere with T cell activation, and largely used in varies autoimmune pathologies [166,167,168,169,170]. Meijer et al. reported significantly reduced conjunctival staining, with no variation in the Schirmer test and tear break up time (TBUT) scores after 2 weeks of treatment with rituximab, in patients with DD associated with SS. Other studies did not report an improvement in objective evaluation after treatment with Rituximab [166].

It could be summarized that different therapeutic options could be considered in relation to the severity of clinical signs and symptoms of DED associated to pSS.

## 5. Conclusions

Our understanding of ocular surface involvement in pSS is improving, based on several studies that have enlightened the molecular pathways at the basis of its pathogenic mechanism. Together with clinical features that are common with other forms of DED, better knowledge of these mechanisms will allow for faster diagnosis and treatment, new methods to evaluate disease severity, and monitoring of disease progression and response to treatment.

In this way, it will be possible to establish early and guided treatment that will allow for better control of the alterations of the ocular surface structures, in order to prevent the evolution towards severe alterations of the quality of life and of vision.

Under these aspects, further studies will definitely help us in achieving such results.
